# Porphyrins as Colorimetric and Photometric Biosensors in Modern Bioanalytical Systems

**DOI:** 10.1002/cbic.202000067

**Published:** 2020-03-30

**Authors:** Karolis Norvaiša, Marc Kielmann, Mathias O. Senge

**Affiliations:** ^1^ School of Chemistry, SFI Tetrapyrrole Laboratory Trinity Biomedical Sciences Institute 152–160 Pearse Street, Trinity College Dublin The University of Dublin Dublin 2 Ireland; ^2^ Institute for Advanced Study (TUM-IAS) Lichtenberg-Strasse 2a 85748 Garching Germany

**Keywords:** Biorecognition, biosensing, colorimetry, detection, porphyrin

## Abstract

Advances in porphyrin chemistry have provided novel materials and exciting technologies for bioanalysis such as colorimetric sensor array (CSA), photo‐electrochemical (PEC) biosensing, and nanocomposites as peroxidase mimetics for glucose detection. This review highlights selected recent advances in the construction of supramolecular assemblies based on the porphyrin macrocycle that provide recognition of various biologically important entities through the unique porphyrin properties associated with colorimetry, spectrophotometry, and photo‐electrochemistry.

## Graphical abstract

**From the** simple colorimetric detection of physiological pH range to complex DNA strand analysis ‐ the versatility of the macrocyclic system and a plenitude of chemical modifications have allowed the production of a plethora of sensitive and selective porphyrin‐based bioreceptors. In this review, we discuss recent advances in modern porphyrin‐based bioanalytical systems in the fields of colorimetry, spectrophotometry and photo‐electrochemistry.

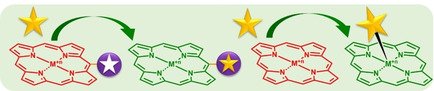

## 1 Introduction

Porphyrins are a unique class of intensely colored pigments, naturally found as biologically active compounds in living organisms where they play an important role in metabolism. Some of the best‐known examples of porphyrins are heme (the red blood cell pigment responsible for oxygen transport) and chlorophyll (the green plant pigment responsible for light‐harvesting and photosynthesis). The diversity of porphyrin functions is, in part, due to the variety of metal ions that bind in the core of the ring system. It was not long after the structure of heme was determined^1^ that porphyrins were noticed by scientists as excellent hosts for metal ions^2^ and well tunable macrocyclic systems.^3^ Porphyrin‐based compounds found applications ranging from photodynamic therapy (PDT),^4^ as dyes or catalysts, to molecular electronic devices for the conversion of solar energy,^5^ and recently as biosensors in modern bioanalytical systems.^6^

**Figure 1:**
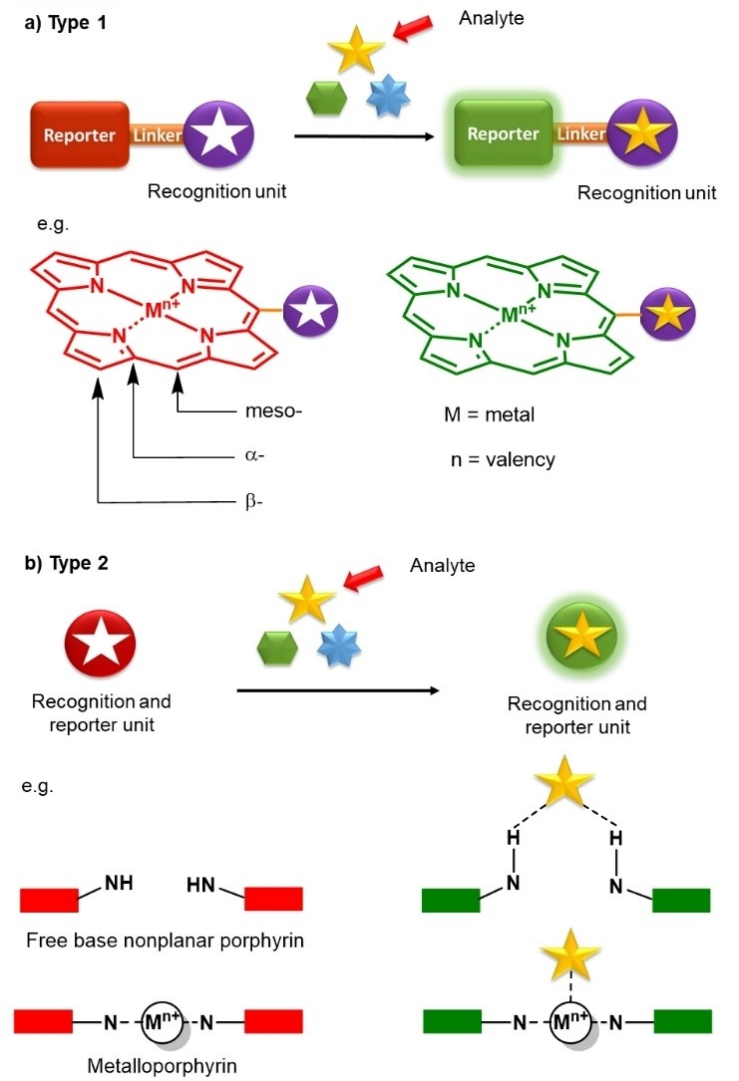
Schematic design of two commonly used organic frame‐based sensing platforms.^20^ a) Recognition and reporter units separated by a linker, e. g., Cd^2+^ binding reported by Lv et al. to the periphery of H_2_‐**7** 26 b) Recognition and reporter function as one unit, e. g., free‐base porphyrin *o*‐H_2_OET_NH2_PP binding pyrophosphate in its inner core reported by Norvaiša et al,^33^ or metalloporphyrin Ni^II^TPP(CN)_4_ coordinating CN^‐^ anions to a metal center reported by Kumar et al.28b

Modern biosensing approaches usually involve five components:^7^ 1) the analyte, a substance of interest that the biosensor is designed to detect; 2) the bioreceptor, a molecule consisting of recognition and reporting units. In a binding event, the analyte attaches to a specifically designed site of the bioreceptor (recognition unit) for selective detection. During this process, the reporter unit produces changes in physical properties, giving a measurable signal that is usually proportional to the quantity of analyte‐bioreceptor interactions; 3) a transducer, an element that converts a physical signals during the biorecognition event into a variation in electric parameters; 4) electronics devices, processing units for the transduced signal that is emitted by the reporter unit; and 5) a display, a combination of hardware and software that generates and outputs a signal that can be read by the user, which can be in numeric, graphic, tabular, or in image form. Due to the excellent photo‐ and electro‐sensitive properties, as well as the vast possibilities of chemical modifications available,^8^ porphyrins in bioanalytical systems can function as dual (recognition and/or reporter) components (Figure 1).

“Porphyrin” is derived from the Greek word “porphura”, meaning purple,^9^ and it was later recognized that the intense porphyrin colors originate from a highly conjugated π system. The electronic absorption spectra of porphyrins consist of two distinct regions: the strong intensity Soret or B bands at 380–500 nm and a weaker set of Q bands in the range of 500–750 nm.^10^ The absorption bands lie in the visible region of the electromagnetic spectrum, well within the range of solar radiation. Thus, charge or exciton transfer processes, as in the case of chlorophylls, reduced magnesium porphyrins (i. e., chlorins), play an essential part in chemical energy production and storage.^11^ Porphyrins are thus classified as the “pigments of life”,^12^ and indeed, life as we know it would not be possible without the biological role of porphyrins and their derivatives on Earth.

**Figure 2:**
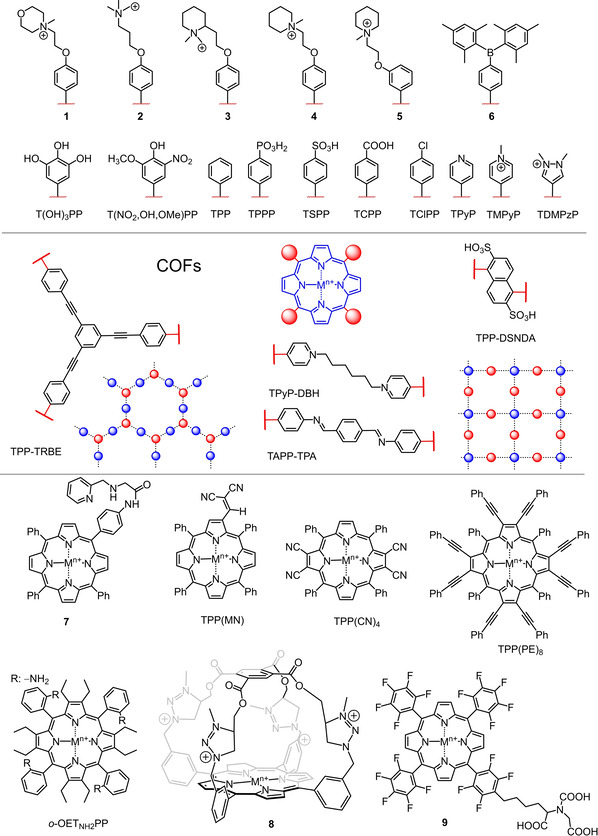
Illustration of porphyrins discussed in this review. The M in the porphyrin core represents a coordinating metal ion, its valency (*n*+) and axial ligands (if any) or the free base form (M=2H).

Porphyrins consist of four heterocyclic pyrrole rings connected via methine bridges, creating a global aromatic system. There are three distinct types of carbon atoms present in the porphyrin structure, C_a_ (α positions) and C_b_ carbon atoms (β positions), as well as methine‐bridged meso positions (C_m_; Figure 1a). The functionalization of these positions and a plentitude of metal coordination reactions to the inner core system have provided opportunities for the design of a variety of porphyrin‐based biosensors. In this review, we will discuss the development of porphyrins as biosensors in modern bioanalytical systems in the last decade. For conciseness, porphyrin‐based electrochemical biosensing is outside the scope of this review; all the discussed porphyrins herein are shown in Figure 2.

## 2 Porphyrins in Bioanalytical Systems

The detection of environmental pollutants or biologically important entities are of crucial importance in order to identify, quantify, or appropriately dispose of the corresponding contaminants.^13^ Chemical sensing^14^ provides essential information that allows monitoring analytes of industrial, environmental, or medicinal relevance, to name but a few examples.^15^

In recent years, porphyrins have emerged as an important class of sensors^16^, ^17^, ^18^ that are used in a large capacity to detect volatile organic compounds (VOCs), reactive oxygen species (ROS), toxic industrial chemicals, metal ions, explosives, pathogens, etc.^19^ Selective recognition can be achieved by supramolecular interactions or through covalent bond formation/cleavage. To employ systems for the selective recognition of compounds, clearly detectable changes in the physical or chemical properties must occur. Thus, substrate binding and reporter function are two main “adjusting screws” in small organic molecule‐based biosensors.

The molecular design of porphyrin‐based sensors can be classified into two distinct types, as described by Ding et al. (Figure 1).^20^ In Figure 1a, the probe's organic framework is made up of the recognition unit that selectively interacts with the target substrate and the reporter unit which photophysically indicates analyte interaction with the receptor. This is like the allosteric regulation of an enzyme (Type 1). In some cases, a linker between these groups is required to control electron flow, or energy transfer processes.

In comparison, Type 2 (Figure 1b), consists of only one component that is responsible for both the recognition and reporting function. This is exemplified by N−H⋅⋅⋅X‐type3e coordination of analytes in the core system of the nonplanar porphyrins or ligation by the central metal in metalloporphyrins. Here, we will give a brief overview on these two types of porphyrin‐based sensing platforms, which rely on colorimetric, spectroscopic, or photo‐electrochemical responses.

### 2.1 Colorimetry

Colorimetry is one of the oldest analytical techniques, which stretches back even before the beginnings of chemistry, with straightforward “naked‐eye” qualification. Color indication is an attractive approach for guest reporting due to its simplicity and general applicability in conventional visual sensing, independent from expensive and complicated equipment. The molecular design for a colorimetric response depends and varies on a selection of chromophores. As porphyrins have an extended conjugated macrocyclic system and thus, are vibrant in color, a range of corresponding intensely colored detectors has been studied.^18^ The colorimetric responses to the corresponding analytes of the discussed porphyrins in this paragraph are presented in Table 1.

**Table 1:** Colorimetric responses to various analytes of the porphyrins discussed.

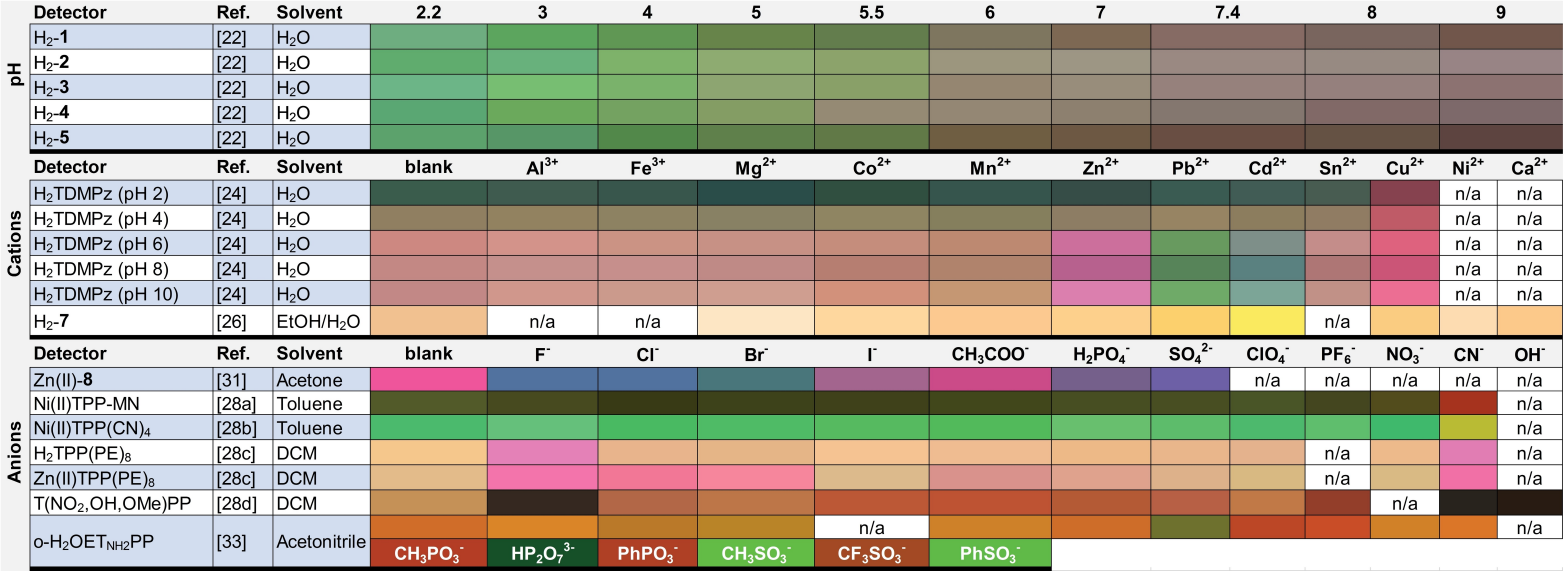

Let us begin with pH sensors. The normal range of the physiological pH value is between 7.36 and 7.44. Precise and simple monitoring of this range is in high demand. For example, in a tumor microenvironment, the pH can drop to as low as 5.5.^21^ A pH sensing platform for the physiological range by a sole fluorophore was reported using a group of five water‐soluble porphyrins (H_2_ **1–5**) showing pH‐dependent colorimetric and fluorescence behavior.^22^ These bimodal compounds were successfully used as pH sensing probes in living cells. Similarly, the water‐soluble porphyrin H_2_TDMPzP was noted to be an excellent colorimetric pH sensing probe. Moreover, selective colorimetric response of H_2_TDMPzP to various metals was observed in different ranges of pH. Cu^2+^ was detected in the pH range of 2–4, while Zn^2+^, Pb^2+^, Cd^2+^ were sensed in the pH range of 6–10. This colorful multimodal porphyrin derivative was later developed into a microfluidic paper‐based analytical device (μPAD)^23^ for practical application in drinking water analysis.^24^ Cadmium is an environmentally concerning highly toxic metal that can cause serious health issues even at trace amounts of exposures.^25^ To monitor its presence, selective naked‐eye detection of Cd^2+^ was reported by Lv et al. In the event of Cd^2+^ binding, the porphyrin‐based sensing probe H_2_‐**7** showed a clear colorimetric response in the pH range of 6.0–8.5.^26^

**Figure 3:**
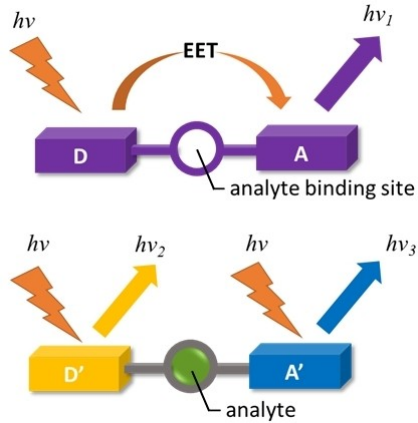
Schematic representation of fluoride sensing reported by Swamy et al.^30^ Binding of the analyte disrupts electronic communication linked between donor and acceptor resulting in an altered fluorogenic response.

Other in‐depth studies on porphyrins as naked‐eye detectors for various analytes were conducted by Sankar and his group, with the focus on the detection of toxic anions,^27^ cyanide,^28^ and the physiologically important fluoride.^29^ The colorimetric diversity upon coordination of the analytes is represented by the synthesized porphyrins, Ni^II^TPP(MN), Ni^II^TPP(CN)_4_, H_2_TPP(PE)_8_, Zn^II^TPP(PE)_8_, and nitrovanillin porphyrin (H_2_T(NO_2_,OH,OMe)PP).^28^ A dual‐function, peripherally triarylborane‐decorated porphyrin Zn^II^‐**6**, reported by Swamy et al., acted as a bifunctional receptor for F^−^ and CN^−^ recognition.^30^ While photophysical studies indicated cyanide binding to the Zn^II^ center, exposure to fluoride anions resulted in cessation of the electronic energy transfer (EET) between the borane and porphyrin units, leading to visual and fluorogenic responses (Figure 3). Supramolecular structures like Zn^II^‐containing positively charged triazoliumporphyrin cages (Zn^II^‐**8**) presents purple to blue color transitions depending on the encapsulation properties of various analytes.^31^ Distinct color variations in the presence of fluorides, chlorides, and oxyanions highlight the solvatochromic properties of the caged system.

As seen in the previous examples, most porphyrin‐based colorimetric probes are either metal‐bearing porphyrins (metalloporphyrins) capable to axially coordinate the targeted analytes, systems that have a distinct bridged binding site, or platforms that contain groups which can perturb the macrocyclic aromaticity. An example for the latter are peripheral hydroxyphenyl moieties for switching between porphyrinoid states and porphyrin in the presence of certain analytes.^32^ However, we have recently demonstrated that highly nonplanar, dodecasubstituted porphyrins with peripheral coordinating groups (*o*‐H_2_OET_NH2_PP) can bind pyrophosphate in its inner core system and displace other weakly coordinating substrates without the need for a central metal.^33^ As a result, manipulation of the N−H⋅⋅⋅X‐type coordination to substrates opens another avenue for the development of metal‐free porphyrin‐based colorimetric probes for a wide range of analytes.^18^ In addition, as related systems are organocatalysts; this might allow the development of multifunctional binding, sensing and degrading systems.^34^

**Figure 4:**
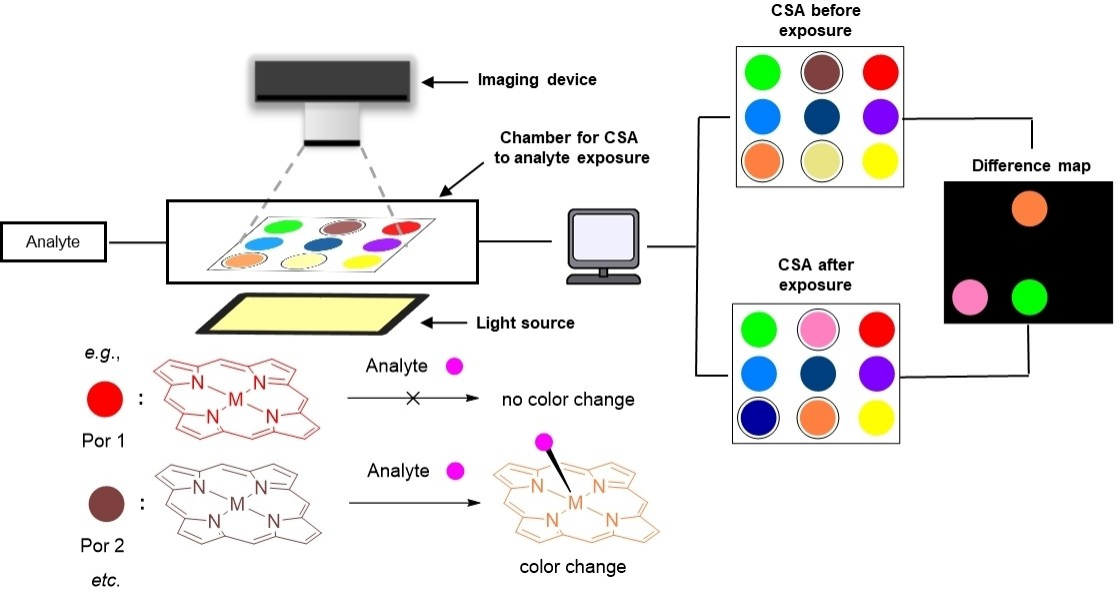
Schematic diagram of a CSA device. Pixel by pixel, the imaging device captures pictures of well‐lighted CSA before and after exposure to the analyte. Later, the computer renders a difference map highlighting the areas of changes in color. This reflects the analyte interacting with the corresponding receptors.

The colorimetric response of porphyrins to a range of analytes is an important feature to be used in optical devices.^35^ One of the fundamental requirements for designing a colorimetric sensor array (CSA) is the chemoresponsive intermolecular interaction with analytes. Naturally, porphyrins and metalloporphyrins are a good choice that shaped the first colorimetric recognition devices, utilizing aspects of Lewis and Brønsted acid‐base interactions of dyes developed by Suslick.^36^ The sensing platforms are made up of various stimuli‐responsive dyes, arranged individual samples in a matrix‐like pattern (i. e., in an *n*×*n* grid similar to a table, Figure 4). These are digitally imaged with respect to their color before and after exposure to the analyte. The imaging can be performed using ordinary flatbed scanners or digital cameras. From the obtained images, pixel by pixel, a difference map is generated using differences in RGB (red green blue) values, highlighting the activity of each dye sensor.

As previously shown, porphyrins have excellent chromatic properties, resulting in distinguishable optical and therefore, electronic changes before and during interactions with a wide range of analytes. Paolesse and di Natale's pioneering development of porphyrins as opto‐electronic noses (a device used for detection of gases) and tongues (a device used in solution studies) have drawn much attention for designing porphyrin containing sensor devices.^4^, ^37^ Today, optical sensing devices such as CSA offer a simple and effective means for the detection and monitoring of environmentally relevant, toxic materials.

For example, an effective artificial nose for the NH_3_ concentration analysis uses a porphyrin‐containing CSA.^38^ Hou et al. showed a colorimetric artificial tongue for the identification of proteins,^39^ which is of importance for the detection of toxic pathogens.^40^ Extensive work by Gu and co‐workers has produced multiple theoretical studies on CSAs to determine the binding abilities of metalloporphyrins for small VOCs.^41^ The importance of the early diagnosis of patients with lung diseases has introduced multitude of breath analysis methods.^42^ Mei and co‐workers explored the identification of VOCs in breath samples of patients and later fabricated a device composed of a porphyrin‐containing CSA for detection of early stages of lung cancer.^43^, ^44^

Food quality and safety is an important factor for the sustainable development of a healthy society. For example, melamine, due to its high nitrogen content and low cost, is illegally added to milk products to falsify the protein quantity. Yang et al. proposed a visualized sensor array approach containing fluorophenyl‐ and sulfonatophenylporphyrins for discriminating between melamine and its analogs.^45^ Public attention to the quality of fish, especially in Asia, due, in part, being the highest consumers of fish in the world, has significantly increased in recent decades. Huang et al. employed a CSA containing ten types of porphyrin to evaluate fish freshness,^46^ and later Lv et al. provided research on VOCs characteristics^47^ and lead content^48^ in fish. The versatility of porphyrin‐containing CSA was also shown to be compatible with the monitoring of various fermentation processes involving yeast,^49^ black tea,^50^ and vinegar.^51^ Porphyrins have even been used as artificial noses to determine the quality of alcoholic drinks, such as baijiu.^52^ As technologies progress, the development of porphyrin‐containing optical probes is advancing. Examples are imprinted composite membrane materials^53^ and miniaturized reflectance devices^54^ for long‐term environmental monitoring, with many more to come.^55^

### 2.2 Spectrophotometry

**Figure 5:**
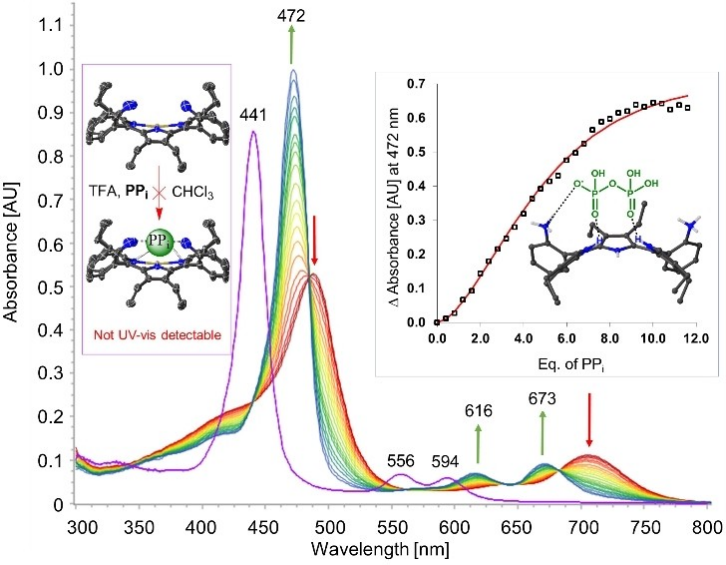
Determination of core interactions through UV‐vis titration.^33^ *o*‐H_2_OET_NH2_PP interacting with pyrophosphate (PP_i_) under acidic conditions

Colorimetric molecular devices have one universal property, absorption in the UV‐vis region of light. This sets UV‐vis absorbance as a powerful method for detailed host‐guest interaction studies. For example, UV‐vis absorbance titration was used to determine binding constants and to estimate the stoichiometry of supramolecular assemblies.^56^, ^57^ A recent example is our study of using dodecasubstituted porphyrins that exhibited gradual absorbance changes in the presence of pyrophosphate. Interestingly, no spectral changes were observed during analyte administration upon Ni^II^ insertion in the inner core system.^33^ This highlighted UV‐vis absorption as a useful tool to conclude analyte to porphyrin core interactions (Figure 5). As it is well known that macrocycle deformations can modulate porphyrin basicity,^58^ dodecasubstituted porphyrins are excellent targets for monitoring pH. Vinogradov and co‐workers showed highly nonplanar dendritic porphyrins with appended hydrophilic groups as a photometric pH probe in aqueous solutions.^59^

**Figure 6:**
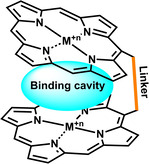
Schematic representation of “Tweezer‐like” porphyrin dimers.

Porphyrin dimers are an emerging and prospective field for analyte detection.^60^ As demonstrated by Borovkov and co‐workers “tweezer‐like” (Figure 6) porphyrin dimers can form 3D cavities between the two subunits by stacking or repelling effects, and these voids may host, for example, histidine,^61^ urea,^62^ aromatic amines,^63^ acetone and ammonia^64^ which coordinate to the porphyrin core, resulting in spectroscopic changes. Similarly, a 3,5‐bisporphyrinylpyridine derivative reported by Moura et al. served as an effective fluorescent probe for a multitude of metals, such as Zn^2+^, Cd^2+^, Hg^2+^, and Cu^2+^.^65^

Recently, solid‐state thin‐film colorimetric vapor sensors acting as so‐called “electronic noses” (chemical sensors, connected to a pattern‐recognition system) have attracted scientists due to the straightforward use of UV‐vis spectrometers with very little modification. Various techniques were used in generating thin porphyrin films, for example, by Langmuir‐Blodgett (LB),^66^ Langmuir‐Schaefer (LS),^67^ spin coating,^68^ or immobilization on reverse‐phase silica gel.^69^ This allowed large‐scale and quick gas analysis of alcohol vapors^68^ or VOCs.^66^, ^69^ Hence, solid‐state porphyrin probes may have the potential to be used as simple breath analysis tools in the near future.42b Moreover, the cost of optical biosensing might tremendously decrease and be readily available as part of integrated design in “everyday” devices, such as computer cameras, cell phones, etc.^70^

The sensitivity of UV‐vis absorbance originating from chromophores is rather limited to proton transfer, tautomerism, skeletal isomerism, charge‐transfer, or polarization. On the other hand, fluorescence is much more sensitive to geometrical and electronic changes. In addition to all of the processes exhibited by the chromophore, fluorescence changes can also be monitored by solvent displacement, conformational restrictions, quenching by guests, or disruption of electronic communication, which give rise to a useful, very sensitive tool for analyte detection.^71^

Some of the most iconic fluorescence sensors were modified to switch between an on and off state of the fluorescence as a design principle. For example, Prabphal et al. reported H_2_TMPyP as a fluorescence turn‐off sensor when complexed with Cu^2+^,^72^ whereas Jiang et al. introduced the turn‐off state of H_2_TMPyP‐oxTMB (3,3′,5,5′‐tetramethylbenzidine diimine) complex as a sensitive fluorescence turn‐on sensor for glutathione (GSH).^73^ Slow addition of GSH disrupts quenched form of H_2_TMPyP‐oxTMB complex, enhancing porphyrin fluorescence for indirect GSH monitoring. A recent review by Garcia‐Sampedro et al. highlights the versatility of the simple and commercially available H_2_TMPyP in biomedical applications.^74^

The ability to accurately measure molecular oxygen in biological samples containing respiring cells and tissues is critical for further advances in medicine. O_2_ is a key metabolite in mammalian cells producing energy‐rich adenosine triphosphate (ATP) molecules, and vital for numerous enzymatic reactions.^75^ Low cellular oxygen levels, known as hypoxia, can cause serious health issues and promote tumor growth.^76^ Moreover, the efficiency of cancer treatment by photodynamic therapy (PDT) is dependent on the availability of tissue oxygen which can be depleted during the generation of reactive oxygen species (ROS).^5^ One of the most important functions of porphyrins in PDT is its capability to produce these ROS to induce cell death in PDT, stimulate immune responses or to promote anti‐angiogenesis.^77^

Also, recall that oxygen is supplied and stored by a family of Fe^II^‐containing porphyrins, the hemes. Hence, porphyrins as oxygen‐sensitive materials, are amongst the most popular O_2_ molecular sensors. Due to limitations (e. g., short lifetime, constant need to renew of electrolyte, loss of oxygen) in electrochemical oxygen sensing,^78^ luminescence‐based sensors have attracted more attention in recent years. At the cellular level, O_2_ is usually found in mitochondria and propagates towards the plasma membrane.

Optical imaging of oxygen in biological systems by porphyrins have been well studied by Vinogradov and co‐workers.^79^ Fercher et al. investigated various Pt^II^‐containing coproporphyrin derivatives for sensing of intercellular oxygen through phosphorescence quenching; however, it was found that the self‐loading into mammalian cells was rather inefficient.^80^ Hence, cell‐penetrating derivatives of (coproporphyrinato I)platinum(II) complexes covalently linked to positively charged peptides were prepared and allowed the monitoring of local oxygen levels distributed in cytoplasm and mitochondria measured by a time‐resolved fluorescent reader.^81^

Later, Pt^II^‐containing porphyrin derivative Pt^II^‐**9** bearing nitrilotriacetate (NTA) and His‐containing peptide conjugates was successfully employed for intracellular loading and phosphorescence quenching‐based sensing of oxygen.^82^ This research highlighted the prospect of biomaterials labeled with phosphorescent platinum porphyrins for a wide range of intercellular analyses. Furthermore, studied iridium porphyrins with axial ligands bearing cell‐penetrating and tumor‐targeting peptides allowed probing of intercellular oxygen; however, the moderate photostability limited its practical use for O_2_ imaging.^83^ Intercellular oxygen sensing using porphyrins is a potential area for the development of oxygen‐compensated glucose‐monitoring optodes^84^ and simultaneous insulin infusion.^85^ In addition to oxygen sensing, the use of cell‐penetrating porphyrin‐based fluorophores is promising for monitoring physical damages to the cell membrane.^86^

### 2.3 Photo‐electrochemistry

**Figure 7:**
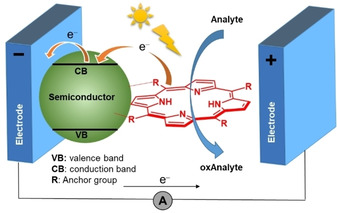
Schematic illustration of a PEC biosensor.

Strong absorbance of visible light can generate excited electrons that can be pumped into semiconductors forming photo‐induced electronic devices. In fact, porphyrins have been extensively studied for use in dye‐sensitized solar cells (DSSC),6c organic solar cells (OSC),6a, 6b, 6d and most recently photo‐electrochemical (PEC) biosensing.^87^ In PEC biosensing, interactions between the analyte and a dye receptor upon light irradiation are detected by changes in the photocurrent signal. The universal design of a basic PEC biosensing platform consists of electrodes (e. g., indium tin oxide (ITO), Pt, Au, etc.) modified with semiconductor nanoparticles (NPs), such as ZnO, ZrO_2_, TiO_2_ and more anchored with a light‐sensitive dye, e. g., a porphyrin. Upon photoexcitation, the porphyrin molecules exhibit ultrafast electron injection into the conduction band (CB) of the semiconductor while the analyte acts as a sacrificial electron donor to scavenge the photogenerated electron holes located on the excited state of the porphyrin. Photoinduced chemical transformation of the analyte, for example oxidation, enhances the photocurrent signal that produces an amperometric response (Figure 7).

An excellent example of PEC biosensing was reported by Tu et al. using TiO_2_ nanoparticles decorated with Fe^III^ClTSPP for glutathione (GSH) sensing^88^ and ZnO−H_2_TCPP composite for the detection of cysteine.^89^ Zinc oxide provides the possibility to fabricate ordered nanostructures with high surface/volume ratios. This was successfully shown by Magna et al., who combined ZnO nanorods (NRs) and porphyrins as an effective PEC sensor for gas‐phase VOCs.^90^ GSH, as a biochemical antioxidant plays an important role in various metabolic processes, maintains the necessary redox balance in cells and actively participates in plant phytoremediation.^91^ Zhu et al. presented on/off detection for glutathione using a nanocomposite consisting of calcium montmorillonite decorated with porphyrin‐functionalized titanium dioxide (H_2_TCPP−TiO_2_−MMT).^92^ A more complicated PEC immunosensor system introduced by Shu et al. was based on a H_2_TSPP−TiO_2_ composite.^93^ The photocurrent response was supported by the affinity of carcinoembryonic antigen (CEA) to H_2_TSPP−TiO_2_. Likewise, the evaluation of cell‐surface carbohydrates with a TiO_2_‐modified PEC biosensor carrying porphyrin‐appended boronic acid complexes to the cell membrane was proposed.^94^

Organic semiconductors play an important role in the modification of PECs. A conventional semiconductor layer was replaced with C_60_, fabricating a PEC biosensor for CEA.^95^ Reduced graphene oxide (RGO) loaded with Au NPs and chitosan (CS) as a RGO−Au−CS semiconductor layer anchored with Zn^II^TCPP was used to monitor hydroquinone.^96^ Zirconium‐based porphyrinic metal‐organic frameworks (MOF) were employed for sensing of dopamine by induced photocurrent response.^97^ Enhancement of the photocurrent intensity was observed when a 1‐naphthalenesulfonate anion‐decorated ITO electrode while subjected to a single‐stranded oligonucleotide prior to H_2_TMPyP adsorption. This resulted in the recognition of oligonucleotides and enzymatic reactions in homogenous solution.^98^

Signal amplification strategies by manipulating porphyrin complexation with DNA have become a potent tool for biorecognition events;^99^ for example, by using a Fe^III^TMPyP−DNA−AuNPs probe on a gold electrode^100^, ^101^ or a Fe^III^TMPyP^100^−DNA−CdS complex.^102^ The resulting PEC biosensors exhibited good performance for the detection of DNA, based on catalytic activity of porphyrin. Another example, is the monitoring of microRNA‐141 by the formation of a DNA “super‐sandwich” structure on the electrode surface for loading manganese(III) protoporphyrin IX.^103^ From simple biologically important entities to complex polynucleotide monitoring, photo‐electrochemical biosensing using porphyrins is a prospective and quickly developing field of research.

## 3 Macromolecular Recognition and Devices

### 3.1 Carbohydrate monitoring

Among many biological carbohydrates found in nature, glucose is arguably one of the most prominent. As the primary fuel for aerobic and anaerobic respiration, the energy potential stored in glucose downstream pathways is responsible for growth and reproduction. In plants, glucose can be found in forms of starch, sucrose, cellulose and amylose. In foods, particularly those that are plant‐derived, high levels of glucose are present. Deviation from the normal range of 80–120 mg/dL (4.4–6.6 mM) glucose in the blood can cause diabetes mellitus (hyperglycemia or insulin deficiency) which is a leading cause for death and disabilities in the world. Therefore, monitoring of the glucose level is an important analytical task, as over 40 % of all blood sample tests taken are related to diabetes.^104^

**Figure 8:**
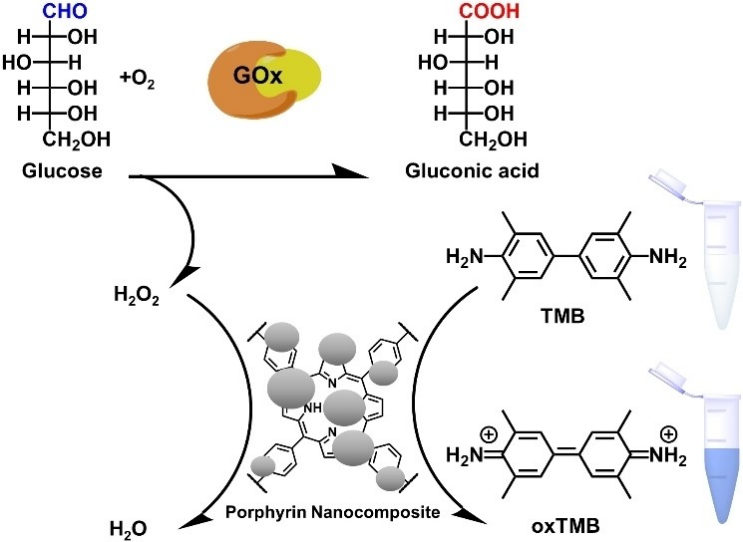
General schematic illustration of a colorimetric sensor for glucose detection with a glucose oxidase (GOx)‐ and porphyrin nanocomposites‐catalyzed reaction.^105^

In recent years, a multitude of methods and glucose biosensors have been developed.^104^ One of these is an optical glucose biosensor mimicking peroxidase activity.^105^ Glucose reduction to gluconic acid by glucose oxidase (GOx) produces H_2_O_2_ that is used to catalyze the conversion of TMB (3,3’,5,5’‐tetramethylbenzidine) by a metal‐bearing material to form the bright blue color of oxTMB (3,3′,5,5′‐tetramethylbenzidine diimine) (Figure 8). The intrinsic peroxidase activity possessed by magnetic Fe_3_O_4_ nanoparticles was first discovered by Gao et al.^106^ Hybrid composites with porphyrins have been extensively explored, showing combined properties of the individual components and performance enhancement.^107^ Hence, porphyrins play an important role in the design of nanocomposite materials used for colorimetric glucose biosensing (Table 2).

**Table 2:** Comparison of the kinetic data for peroxidase‐like behavior studies on porphyrin‐based nanocomposites and other catalysts.

Porphyrin	Composite material^[a]^	Type^[b]^	Glucose	TMB		H_2_O_2_			Ref.
			LOD^[c]^ [μM]	*K*_m_^[d]^ [mM]	*v*_max_^[e]^ [×10^−8^ M s^−1^]	LOD^[c]^ [μM]	*K*_m_^[d]^ [mM]	*V*_max_^[e]^ [×10^−8^ M s^−1^]	
	Fe_3_O_4_	NPs	n.a.	0.098	3.44	n.a.	154.00	9.78	106
H_2_TCPP	Fe_3_O_4_	NPs	2.21	0.439	19.08	1.07	0.919	1.075	108
H_2_TCPP	NiO	NPs	20	0.011	48.2	8	39.10	1.38	109
H_2_TCPP	CeO_2_	NPs	0.19	0.085	435	n.a.	0.254	1.31	110b
H_2_TCPP	CeO_2_	NRs	33	0.011	26.9	6.1	0.366	0.496	110a
H_2_TClPP	CuFe_2_O_4_−SiO_2_	NPs	3.62	n.a.	n.a.	1.015	n.a.	n.a.	116
H_2_TCPP	CdS	NPs	7.02	0.072	0.4369	46	2.000	0.173	113
H_2_TCPP	γ‐Fe_2_O_3_	NPs	2.54	0.026	0.017	1.73	0.013	2.14	112
H_2_TCPP	Co_3_O_4_	NPs	86	0.028	0.64	40	6.100	0.7	111
H_2_TCPP	ZnFe_2_O_4_	NPs	5.5	0.026	2.88	0.86	0.045	1.4	115
H_2_TCPP	ZnS	NPs	36	0.055	0.048	15.8	0.172	10.05	114
Fe^III^ClTCPP	PCN‐224	MOF	22	0.030	34.2	1.6	n.a.	n.a.	119
Fe^III^ClTCPP	PCN‐222	MOF	2.2	0.005	18.8	1	0.097	10.00	118a
Fe^III^TBrPP	TRBE	COF	3	0.064	1.94	6.5	133.00	2.12	121
Fe^II^TAPP	TPA	COF	1	0.020	3.83	1.1	0.143	4.74	122
Fe^III^ClTPP	DSNDA	COF	16.38	0.047	1.17	26.7	1.490	19.4	123
Fe^III^TPyP^100^	DBH	COF	0.098	0.106	n.a.	n.a.	4.870	n.a.	124
Fe^III^TPyP^100^			n.a.	0.130	n.a.	n.a.	6.020	n.a.	124

[a] Nanocomposite linking material used with porphyrin. [b] Type of nanomaterial. [c] Limit of detection. [d] Michaelis constant. [e] Maximum rate of conversion.

Extensive work by Liu and co‐workers explored various porphyrin‐based nanocomposite materials as peroxidase mimics. Therein, the key component was the porphyrin H_2_TCPP, used to functionalize metal oxides Fe_3_O_4_,^108^ NiO,^109^ CeO_2_,^110^ Co_3_O_4_,^111^ and γ‐Fe_2_O_3_,^112^ the metal sulfides CdS and ZnS,^114^ and ZnFe_2_O_4_, respectively. Moreover, SiO_2_ nanospheres were used as a support to anchor the magnetic binary metal oxide CuFe_2_O_4_ to H_2_TClPP in order to obtain a H_2_TClPP−CuFe_2_O_4_−SiO_2_ nanocomposite.^116^ Another example by Yang et al. demonstrated peroxidase‐like activity of Fe^III^ClTPPP covalently attached to gold nanoroads (NRs), forming a Fe^III^ClTPPP−Au_2_S−AuAgS nanocomposite.^117^

Most recently, well‐ordered structures similar to porphyrin‐based MOFs have drawn considerable attention as a substitute for conventional metal oxide‐based nanostructures due to the high concentration of active sites. For example, incorporation of Fe^III^ClTCPP into zirconium clusters was investigated, forming uniform nanoparticles such as PCN‐222^118^ or PCN‐224^119^ as efficient nanostructures for the detection of glucose. Just like MOFs, covalent organic frameworks (COF)^120^ have a large specific surface area, tunable pore structures, and high thermal, chemical, and water stability. This has motivated the construction of 2D‐polymeric nanostructures (Figure 2), such as Fe^III^ClTPP−TRBE,^121^ Fe^II^TPP−TPA,^122^ Fe^III^ClTPP−DSNDA,^123^ and nanocapsules like Fe^III^TPyP−DBH.^100^, ^124^ Even haemin encapsulated by a protein nanocage (haemin‐SR1) showed successful oxidation of TMB for the detection of glucose.^125^

Clever design of porphyrin‐based COFs could potentially serve in different sensing platforms, for example, for fluorescence turn‐on biosensing of aminoglycoside,^126^ and encapsulated by biocompatible and biodegradable carriers^127^ or hydrogels^128^ to be studied *in vivo*. Hopefully, soon, porphyrin‐based continuous monitoring devices will play an essential role in regulation of glucose levels^84^ and simultaneous injection of insulin^85^ while reducing the effects of diabetes‐related diseases across the world.^129^

### 3.2 Protein‐based detection

Proteins are the “working machinery” of life, with functions ranging from energy storage and metabolism to the regulation of cellular functions.^130^ Heme‐containing proteins like hemoglobin^131^ and cytochrome P450^132^ have recently found use as biosensors, and the effective heme‐based detection of small analytes, such as O_2_,^133^ CN^−^,^134^ NO,^135^ histidine,^136^ glucose,^125^ and uric acid.^137^ This could further be expanded to more complex systems, such as protein detection^138^ and functional control,^139^ along with recognition and inhibition of bacteria,^140^ parasites,^141^ and cancer cells.^142^

Porphyrin‐guest complexes were proven to act as sensitive probes for amino acids, for example, using coordination complexes of cobalt ions and bisporphyrins.^143^ At the same time, porphyrins found its way as mimicking a receptor or surface blocking agents for potential role in protein labeling. For example, Mn^III^ClTPP^144^ and Sn^IV^Cl_2_TPP^145^ were studied as promising protein labeling tools due to the range of analytical methods they enable. The detection and monitoring of unique proteins^146^ can indicate protein activities in organisms or cells.^147^ Yang et al. showed that H_2_TSPP can act as an optical probe in detection of 2‐hydroxyquinoxaline, a biomarker of plant esterase, in inhibition studies using organophosphorus pesticides,^148^ while peripheral modifications of H_2_TC_1_PP with antimicrobial peptides could serve as indicators for bacteria, as presented by Johnson et al.^149^

### 3.3 Polynucleotide recognition and aptasensors

DNA is a fundamental polynucleotide for storage, duplication, and realization of genetic information.^150^ It is not surprising that there is a growing interest in DNA recognition and structural designs containing nucleic acids. Porphyrins are of immense interest in DNA analysis due to well established porphyrin – nucleic acid interactions.^151^ For example, Vaishnavi et al. conducted research on H_2_TMPyP deposed on the surface of negatively charged thioglycolic acid (TGA)‐capped CdTe quantum dots, which show strong fluorescence quenching. However, once in the presence of polynucleotides, H_2_TMPyP binds to double‐stranded DNA (dsDNA) due to the stronger affinity, restoring the fluorescence to CdTe−TGA.^152^

**Figure 9:**
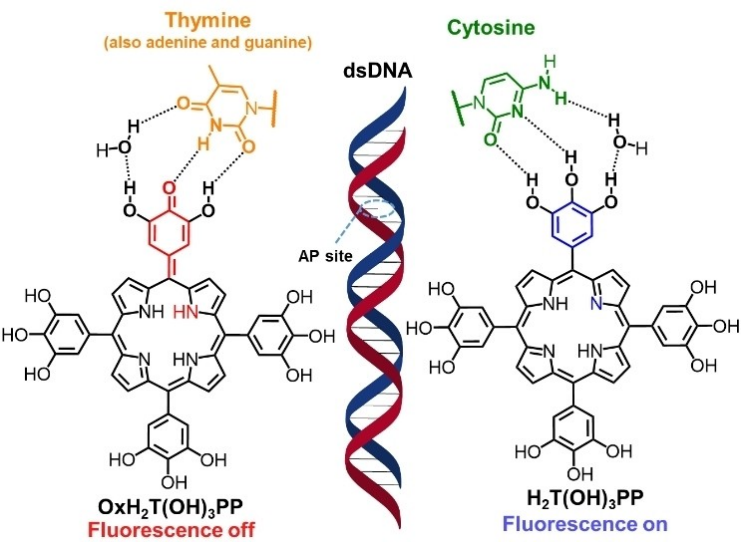
AP site interactions of dsDNA and H_2_T(OH)_3_PP: fluorescence changes are induced upon interaction with cytosine (“on”) and other nucleobases (“off”), respectively.^153^

More detailed DNA analysis was performed by Lin et al., who showed that the identification of one target cytosine in a strand of nucleotides could be accomplished once bound with H_2_T(OH)_3_PP. The nonfluorescent porphotrimethene form OxH_2_T(OH)_3_PP “switched on” by conversion to the aromatic porphyrin form by selectively hydrogen bonding to the target cytosine site in dsDNA (Figure 9). The recognition of nucleic acids can only be targeted in the abasic site (AP) and in a particular pH range.^153^

The high affinity of porphyrins to DNA can even be used to discriminate condensed left‐handed Z‐DNA in the presence of canonical right‐handed B‐DNA. The pioneering work on cationic and anionic porphyrins acting as chiroptical probes for Z‐DNA detection has been introduced by the Columbia group led by Berova and Nakanishi in collaboration with the group from Catania University by Purrello and D′Urso.^154^ Zn^II^TMPyP154a, 155 or Ni^II^TSPP154b, 156 induces a strong CD signal by association with Z‐DNA in a Z‐DNA/B‐DNA mixture while showing no observable CD response of the B‐DNA complex. Porphyrin‐DNA complexes are particularly useful in mimicking enzyme catalytic activity, for example, Mn^III^TMPyP^100^−dsDNA^157^ and Fe^III^TMPyP−dsDNA,^100^, ^158^ and show promising potential in bioanalysis and other relevant fields.^159^

**Figure 10:**
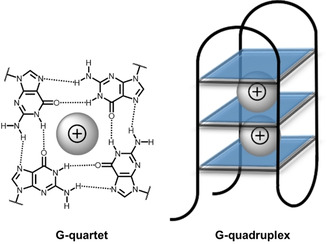
G‐quartets stabilized by Hoogsteen hydrogen bonding and cations between two G‐quartets.

DNA, initially assumed to be a rigid structure responsible only for storing genetic information, is essentially a highly dynamic molecule, capable of forming a number of spatial arrangements.^160^ One of the most widely known structures of DNA is the highly ordered guanine‐rich oligonucleotide sequence known as G‐quadruplex.^161^ G‐quadruplexes are formed by the stacking of planar guanine quartets (G‐quartets) that are composed of four guanine bases arranged in a square‐planar configuration and stabilized via Hoogsteen pairing. The central cavity of G‐quadruplexes is occupied by cations, which neutralize the electrostatic repulsion between guanine oxygen atoms, and thus stabilize the overall structure (Figure 10). G‐quartets form extended three‐dimensional structures through their large aromatic π‐surfaces. Consequently, early ligands in G‐quadruplex‐sensing probes were based on heteroaromatic systems accommodating π–π‐stacking interactions.^162^

Therefore, porphyrin dyes (large heterocyclic, aromatic, highly conjugated systems) binding to G‐quadruplex have been studied since the late 20th century.^163^ For example, studies conducted by Kong and co‐workers on cationic porphyrins showed the ability to specifically recognize nucleic acid G‐quadruplexes over dsDNA and single‐stranded DNA(ssDNA).^164^ These cationic porphyrin‐based G‐quadruplex probes have high potential in pH‐sensing,^165^ DNA logic gates construction,^166^ and cancer targeting/imaging.^99^, ^167^ Out of all naturally occurring porphyrins, hemin complexes with G‐quadruplex have been studied extensively, with a multitude of the reviews present in the literature.^168^ Iron(III)‐containing protoporphyrin IX with an chlorine as axial ligand in coordination with G‐quadruplex has found its applications as peroxidase‐mimicking DNAzyme biosensors,168b, 168d electrochemical signal generators,168e, 168f, 168g ion, ligand, DNA sensors,168a, 168c, 168h, 168j and versatile scaffold for catalysis.168i

Porphyrin dye integration/displacement and (un)folding of G‐quadruplex DNA are the main recognition mechanisms used in G‐quadruplex biosensing. Through these methods, the detection of various cations, for example, Cu^2+^,^169^ Pb^2+^,^170^ and Hg^2+^,^170^, ^171^ small‐molecular ATP,^172^ adenosine,^173^ ʟ‐histidine,^174^ and aflatoxin B_1_ in grape juice^175^ have been developed. Due to the sensitivity of optical porphyrin sensors and their ability to serve as ligands for G‐quadruplexes, the probes could also detect structural changes in proteins^176^ and DNA^177^ and have potential to monitor the activity of enzymes, for example, kinases.^178^

## 4 Summary and Outlook

In this brief review, we focused on porphyrins as biosensors in modern bioanalytical systems, and rather than providing an exhaustive list, we aimed to inform through selecting representative examples of contemporary porphyrin‐based biosensing platforms. The multitude of porphyrin physicochemical properties was discussed and shown to serve as an essential prerequisite to engineer and tune efficient biosensor systems. We elaborated that simple colorimetric responses to pH changes and ions, detectable by naked eye, were evolved into elaborate CSA imaging devices to detect VOCs and to determine the stages of certain diseases. The strong photosensitive nature of porphyrins can not only be applied to substrate/reactant determination, but porphyrins can also perform as efficient energy/electron transfer components in PEC signaling for bioanalysis.

Through various porphyrin modifications, enzyme‐mimicking devices were examined that provide efficient quantification of glucose. Moreover, porphyrins can selectively sense G‐quadruplex over other DNA conformations that are present in a cellular environment, such as ssDNA and dsDNA. These cyclic tetrapyrrole derivatives will likely play an important role in understanding the complex structures and reveal new functions of polynucleotides.

Porphyrins have a brilliant future in the monitoring and detection of biologically and environmentally important entities. Many challenges are yet to be faced, such as the complexity of “real” samples “in the field”; repeatability, reliability, and robustness. Hopefully, the reviewed porphyrin‐based technologies will become useful for clinicians and material scientists, who require methods that allow for the highly sensitive detection of analytes. As such, new work in the design of porphyrin biosensor systems may lead to the development and emergence of unmatched bioanalytical tools and devices.

## Conflict of interest

The authors declare no conflict of interest.

## Biographical Information

*Karolis Norvaiša was born in Kaunas, Lithuania in 1994. He graduated from Kaunas University of Technology in 2017 with a bachelor's degree in Applied Chemistry. Soon after, he received a postgraduate fellowship from the Irish Research Council and joined Prof. Senge's group in Ireland to continue his research as a Ph.D. student in the field of nonplanar porphyrins. His research focuses on crystal engineering and atropisomerism effects in sensing applications. Recently, he joined forces in the EU‐wide H2020 FET‐Open project INITIO, which aims to detect and remove chiral pollutants from the environment*.

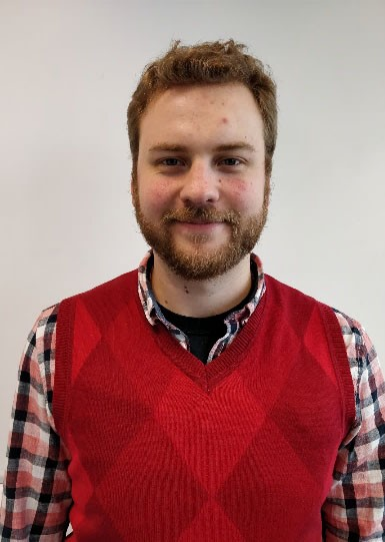

## Biographical Information

*Marc Kielmann studied Chemistry (B.Sc.) and Medicinal and Natural Product Chemistry (M.Sc.) at the Leibniz University, Hannover, working with Profs. Andreas Kirsching and Holger Butenschön. He gained a PhD in the group of Prof. Senge, working on methods development and the synthesis of nonplanar porphyrins for use as organocatalysts and sensors. He stayed as an EU‐funded Postdoctoral Researcher, acting as liaison for the multinational research collaboration INITIO. Marc is currently working as a Scientific Editor for the Beilstein Journal of Organic Chemistry in Frankfurt am Main, and his interests include the ethics of science and open access publishing*.

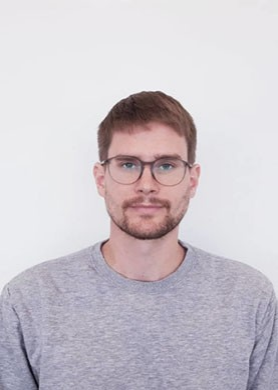

## Biographical Information

*Mathias Senge, studied chemistry and biochemistry in Freiburg, Amherst, Marburg, and Lincoln. After a Ph.D. in plant biochemistry with Prof. Horst Senger (Marburg) and a postdoctoral fellowship with Prof. Kevin M. Smith (UC Davis), he received his habilitation in Organic Chemistry in 1996 at the Freie Universität Berlin. Following a Heisenberg fellowship at the Freie Universität Berlin and UC Davis he was appointed Professor of Organic Chemistry at the Universität Potsdam in 2002, and since 2005 has held the Chair of Organic Chemistry at Trinity College Dublin. His main interests are synthetic organic chemistry, hydrocarbon scaffolds, the (bio)chemistry of tetrapyrroles, photochemistry, ‐biology and ‐medicine, structural chemistry, and history of science*.

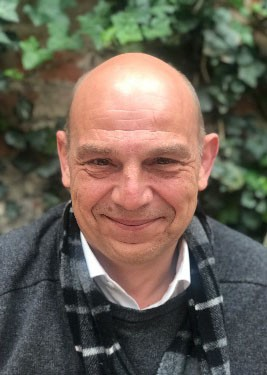

## References

[1] T. K. With , Int. J. Biochem. 1980, 11, 189–200. 6993245 10.1016/0020-711x(80)90219-0

[2a] J. Wojaczyński , L. Latos-Grażyński , Coord. Chem. Rev. 2000, 204, 113–171;

[2b] I. Beletskaya , V. S. Tyurin , A. Y. Tsivadze , R. Guilard , C. Stern , Chem. Rev. 2009, 109, 1659–1713. 19301872 10.1021/cr800247a

[3a] M. O. Senge , T. P. Forsyth , L. T. Nguyen , K. M. Smith , Angew. Chem. Int. Ed. Engl. 1994, 33, 2485–2487;

[3b] M. O. Senge , Chem. Commun. 2006, 243–256; 10.1039/b511389j 16391725

[3c] M. O. Senge , S. A. MacGowan , J. M. O′Brien , Chem. Commun. 2015, 51, 17031–17063; 10.1039/c5cc06254c 26482230

[3d] B. Szyszko , M. J. Białek , E. Pacholska-Dudziak , L. Latos-Grażyński , Chem. Rev. 2017, 117, 2839–2909; 28222606 10.1021/acs.chemrev.6b00423

[3e] M. Kielmann , M. O. Senge , Angew. Chem. Int. Ed. 2019 , 58, 418–441; 10.1002/anie.201806281 PMC6391963 30067890

[4] R. Paolesse , S. Nardis , D. Monti , M. Stefanelli , C. Di Natale , Chem. Rev. 2017, 117, 2517–2583. 28222604 10.1021/acs.chemrev.6b00361

[5] S. Callaghan , M. O. Senge , Photochem. Photobiol. Sci. 2018, 17, 1490–1514. 29569665 10.1039/C8PP00008E

[6a] M. Grätzel , J. Photochem. Photobiol. C: Photochem. Rev. 2003, 4, 145–153;

[6b] T. Higashino , H. Imahori , Dalton Trans. 2015, 44, 448–463; 25381701 10.1039/c4dt02756f

[6c] O. Birel , S. Nadeem , H. Duman , J. Fluoresc. 2017, 27, 1075–1085; 28210924 10.1007/s10895-017-2041-2

[6d] A. Mahmood , J.-Y. Hu , B. Xiao , A. Tang , X. Wang , E. Zhou , J. Mater. Chem. A. 2018, 6, 16769–16797.

[7] N. Bhalla , P. Jolly , N. Formisano , P. Estrela , Essays Biochem. 2016, 60, 1–8. 27365030 10.1042/EBC20150001 PMC4986445

[8a] M. O. Senge , Chem. Commun. 2011, 47, 1943–1960; 10.1039/c0cc03984e 21218237

[8b] S. Hiroto , Y. Miyake , H. Shinokubo , Chem. Rev. 2017, 117, 2910–3043; 27709907 10.1021/acs.chemrev.6b00427

[8c] M. Taniguchi , J. S. Lindsey , Chem. Rev. 2017, 117, 344–535. 27498781 10.1021/acs.chemrev.5b00696

[9] R. A. Smith , J. Chem. Educ. 1998, 75, 420.

[10] M. Gouterman , J. Mol. Spectrosc. 1961, 6, 138–163.

[11a] A. A. Ryan , M. O. Senge , Photochem. Photobiol. Sci. 2015, 14, 638–660; 25683614 10.1039/c4pp00435c

[11b] M. O. Senge , A. A. Ryan , K. A. Letchford , S. A. MacGowan , T. Mielke , Symmetry 2014, 6, 781–843.

[12] A. R. Battersby , Nat. Prod. Rep. 2000, 17, 507–526. 11152419 10.1039/b002635m

[13] Y. Ding , Y. Tang , W. Zhu , Y. Xie , Chem. Soc. Rev. 2015, 44, 1101–1112. 25608833 10.1039/c4cs00436a

[14] P. D. Beer , P. A. Gale , Angew. Chem. Int. Ed. 2001, 40, 486–516; 11180358

[15a] Y. Xie , J. P. Hill , R. Charvet , K. Ariga , J. Nanosci. Nanotechnol. 2007, 7, 2969–2993; 18019127 10.1166/jnn.2007.910

[15b] A. D′Amico , C. Di Natale , C. Falconi , E. Martinelli , R. Paolesse , G. Pennazza , M. Santonico , P. J. Sterk , Expert Opin. Med. Diagn. 2012, 6, 175–185; 23480684 10.1517/17530059.2012.665870

[15c] L. Lvova , C. G. Gonçalves , C. Di Natale , A. Legin , D. Kirsanov , R. Paolesse , Talanta 2018, 179, 430–441. 29310257 10.1016/j.talanta.2017.11.019

[16] D. Monti , S. Nardis , M. Stefanelli , R. Paolesse , C. Di Natale , A. D'Amico , J. Sens. 2009, 2009, 1–10.

[17] M. Ekrami , G. Magna , Z. Emam-djomeh , M. Saeed Yarmand , R. Paolesse , C. Di Natale , Sensors 2018, 18, 2279. 10.3390/s18072279 PMC6069155 30011907

[18] Z. Li , J. R. Askim , K. S. Suslick , Chem. Rev. 2019, 119, 231–292. 30207700 10.1021/acs.chemrev.8b00226

[19] M. Kielmann , C. Prior , M. O. Senge , New J. Chem. 2018, 42, 7529–7550.

[20] T. Ding , W.-H. Zhu , Y. Xie , Chem. Rev. 2017, 117, 2203–2256. 27078087 10.1021/acs.chemrev.6b00021

[21] C. R. Justus , L. Dong , L. V. Yang , Front. Physiol. 2013, 4, 354–363. 24367336 10.3389/fphys.2013.00354 PMC3851830

[22] Y.-Y. Liu , M. Wu , L.-N. Zhu , X.-Z. Feng , D.-M. Kong , Chem. Asian J. 2015, 10, 1304–1310. 25779219 10.1002/asia.201500106

[23] S. T. Phillips , G. M. Whitesides and E Carrilho , Anal. Chem. 2010 , 82, 3–10. 20000334 10.1021/ac9013989

[24] R. Pratiwi , M. P. Nguyen , S. Ibrahim , N. Yoshioka , C. S. Henry , D. H. Tjahjono , Talanta 2017, 174, 493–499. 28738613 10.1016/j.talanta.2017.06.041

[25] R. Hernández-Martínez , I. Navarro-Blasco , Food Control 2012, 26, 6–14.

[26] Y. Lv , L. Wub , W. Shen , J. Wang , G. Xuan , X. Sun , J. Porphyrins Phthalocyanines 2015, 19, 1–6.

[27] F. J. Baud , Hum. Exp. Toxicol. 2007, 3, 13254–13262. 10.1177/0960327107070566 17439922

[28a] M. K. Chahala , M. Sankar , RSC Adv. 2015, 5, 99028–99036;

[28b] R. Kumar , N. Chaudhri , M. Sankar , Dalton Trans. 2015, 44, 9149–9157; 25901694 10.1039/c5dt00937e

[28c] R. Kumar , P. Yadav , P. Rathi , M. Sankar , RSC Adv. 2015, 5, 82237–82246;

[28d] T. A. Dar , M. Sankar , ChemistrySelect 2017, 2, 6778–6783.

[29] K. L. Kirk , Biochemistry of The Halogens and Inorganic Halides, Plenum Press, New York, 1991.

[30] P. C. A. Swamy , S. Mukherjee , P. Thilagar , Anal. Chem. 2014, 86, 3616–3624. 24571811 10.1021/ac500230p

[31] L. C. Gilday , N. G. White , P. D. Beer , Dalton Trans. 2012, 41, 7092–7097. 22561990 10.1039/c2dt30124e

[32] M. K. Chahal , M. Sankar , Dalton Trans. 2017, 46, 11669–11678. 28792058 10.1039/c7dt01158j

[33] K. Norvaiša , K. J. Flanagan , D. Gibbons , M. O. Senge , Angew. Chem. 2019, 131, 16705–16709; 10.1002/anie.201907929 PMC6899560 31412154

[34a] M. Roucan , M. Kielmann , S. J. Connon , S. S. R. Bernhard , M. O. Senge , Chem. Commun. 2018, 54, 26–29; 10.1039/c7cc08099a 29226923

[34b] M. Kielmann , N. Grover , W. W. Kalisch , M. O. Senge , Eur. J. Org. Chem. 2019, 2448–2452.

[35] J. R. Askim , M. Mahmoudi , K. S. Suslick , Chem. Soc. Rev. 2013, 42, 8649–8682. 24091381 10.1039/c3cs60179j

[36] N. A. Rakow , K. S. Suslick , Nature 2000, 406, 710–713. 10963592 10.1038/35021028

[37a] C. Di Natale , A. Macagnano , F. Davide , A. D′Amico , R. Paolesse , T. Boschi , M. Faccio , G. Ferri , Sens. Actuators B 1997, 44, 521–526;

[37b] C. Di Natale , R. Paolesse , A. Macagnano , A. Mantini , A. D′Amico , A. Legin , L. Lvova , A. Rudnitskaya , Y. Vlasov , Sens. Actuators B 2000, 64, 15–21;

[37c] C. Di Natale , D. Salimbeni , R. Paolesse , A. Macagnano , A. D′Amico , Sens. Actuators B 2000, 65, 220–226.

[38] Y. Zhang , X.-G. Luo , K. He , D.-Q. Huo , J. Liu , P. Liu , X.-J. Shi , C.-J. Hou , Water Air Soil Poll. 2012, 223, 2969–2977.

[39] C. Hou , J. Dong , G. Zhang , Y. Lei , M. Yang , Y. Zhang , Z. Liu , S. Zhang , D. Huo , Biosens. Bioelectron. 2011, 26, 3981–3986. 21546237 10.1016/j.bios.2010.11.025

[40] P. B. Crowley , P. Ganji , H. Ibrahim , ChemBioChem 2008, 9, 1029–1033. 18389511 10.1002/cbic.200700736

[41a] H. Gu , X. Huang , L. Yao , E. Teye , Y. Wen , IEEE Sens. J. 2014, 14, 2620–2625;

[41b] X. Huang , H. Gu , L. Yao , E. Teye , Y. Wen , J. Comput. Theor. Nanos. 2014, 11, 2194–2198;

[41c] H. Gu , X. Huang , L. Yao , E. Teye , Y. Wen , Mater. Technol. 2014, 29, 220–226;

[41d] H. Gu , X. Huang , L. Yao , E. Teye , Y. Wen , Anal. Methods 2014, 6, 3360–3364;

[41e] H. Gu , Y. Sun , S. Li , X. Huang , H. Dai , Comput. Theor. Chem. 2016, 1094, 13–16;

[41f] H. Gu , Y. Sun , X. Huang , H. Dai , Sens. Mater. 2017, 29, 77–83.

[42a] C. Di Natale , A. Macagnano , E. Martinelli , R. Paolesse , G. D′Arcangelo , C. Roscioni , A. Finazzi-Agrò , A. D′Amico , Biosens. Bioelectron. 2003, 18, 1209–1218; 12835038 10.1016/s0956-5663(03)00086-1

[42b] C. Di Natale , R. Paolesse , E. Martinelli , R. Capuano , Anal. Chim. Acta 2014, 824, 1–17; 24759744 10.1016/j.aca.2014.03.014

[42c] R. Capuano , A. Catini , R. Paolesse , C. Di Natale , J. Clin. Med. 2019, 8. 10.3390/jcm8020235 PMC6406777 30754727

[43] W. Yu , H. Danqun , H. Changjun , F. Huanbao , Y. Mei , L. Xiaogang , Chem. Res. Chin. Univ. 2014, 30, 572–577.

[44] S. Zhao , J. Lei , D. Huo , C. Hou , X. Luo , H. Wu , H. Fa , M. Yang , Sens. Actuators B 2018, 256, 543–552.

[45] L. Yang , D. Huo , Y. Jiang , C. Hou , S. Zhang , Food Addit. Contam. B 2013, 30, 786–795. 10.1080/19440049.2013.793457 23768006

[46] X. Huang , R. Lv , L. Yao , C. Guan , F. Han , E. Teye , Anal. Methods 2015, 7, 1615–1621.

[47] R. Lv , X. Huang , W. Ye , J. H. Aheto , H. Xu , C. Dai , X. Tian , J. Food Process. Eng. 2019, 42, e12952.

[48] R. Lv , X. Huang , J. H. Aheto , C. Dai , X. Tian , J. Food Process Eng. 2019, 42, e13075.

[49] W. Xu , H. Jiang , T. Liu , Y. He , Q. Chen , Anal. Methods 2019, 11, 3294–3300.

[50] H. Li , B. Zhang , W. Hu , Y. Liu , C. Dong , Q. Chen , J. Food Process. Preserv. 2018, 42, e13348.

[51] Q. Chen , A. Liu , J. Zhao , Q. Quyang , Z. Sun , L. Huang , Sens. Actuators B 2013, 183, 608–616.

[52] Z. Ya , K. He , Z.-M. Lu , B. Yi , C.-J. Hou , S. Shan , D.-Q. Huo , X.-G. Luo , Flavour Fragrance J. 2012, 27, 165–170.

[53] Z. Ya , Q. Linlin , Y. Wei , H. Bin , L. Fengmei , H. Changjun , H. Danqun , F. Huanbao , Chem. Res. Chinese U. 2016, 32, 725–730.

[54] B. J. Johnson , J. S. Erickson , J. Kim , A. P. Malanoski , I. A. Leska , S. M. Monk , D. J. Edwards , T. N. Young , J. Verbarg , C. Bovais , Meas. Sci. Technol. 2014, 25, 095101.

[55] B. J. Johnson , A. P. Malanoski , J. S. Erickson , R. Liu , A. R. Remenapp , D. A. Stenger , M. H. Moore , Heliyon 2017, 3, e00312. 28626804 10.1016/j.heliyon.2017.e00312 PMC5463015

[56] P. Thordarson , Chem. Soc. Rev. 2011, 40, 1305–1323. 21125111 10.1039/c0cs00062k

[57] D. B. Hibbert , P. Tjordarson , Chem. Commun. 2016, 52, 12792–12805. 10.1039/c6cc03888c 27779264

[58] O. S. Finikova , A. V. Cheprakov , P. J. Carroll , S. Dalosto , S. A. Vinogradov , Inorg. Chem. 2002, 41, 6944–6946. 12495329 10.1021/ic0260522

[59] S. Thyagarajan , T. Leiding , S. P. Arsköld , A. V. Cheprakov , S. A. Vinogradov , Inorg. Chem. 2010, 49, 9909–9920. 20882973 10.1021/ic100968p PMC2964403

[60a] G. Giancane , V. Borovkov , Y. Inoue , S. Conoci , L. Valli , Soft Matter 2013, 9, 2302–2307;

[60b] A. Colombelli , M. G. Manera , V. Borovkov , G. Giancane , L. Valli , R. Rella , Sens. Actuators B 2017, 246, 1039–1048;

[60c] P. Mondal , S. P. Rath , Coord. Chem. Rev. 2020, 405, 213117.

[61] S. Bettini , R. Pagano , V. Borovkov , G. Giancane , L. Valli , J. Colloid Interface Sci. 2019, 533, 762–770. 30199832 10.1016/j.jcis.2018.08.116

[62] A. Buccolieri , M. Hasan , S. Bettini , V. Bonfrate , L. Salvatore , A. Santino , V. Borovkov , G. Giancane , Anal. Chem. 2018, 90, 6952–6958. 29727561 10.1021/acs.analchem.8b01230

[63] G. Giancane , V. Borovkov , Y. Inoue , L. Valli , J. Colloid Interface Sci. 2012, 385, 282–284. 22858402 10.1016/j.jcis.2012.06.081

[64] A. Buccolieri , D. Manno , A. Serra , A. Santino , M. Hasan , V. Borovkov , G. Giancane , Sens. Actuators B 2018, 257, 685–691.

[65] N. M. M. Moura , C. Núñez , S. M. Santos , M. A. F. Faustino , J. A. S. Cavaleiro , F. A. A. Paz , M. G. P. M. S. Neves , J. L. Capelo , C. Lodeiro , Chem. Eur. J. 2014, 20, 6684–6692. 24782336 10.1002/chem.201402270

[66] A. D. F. Dunbar , S. Brittle , T. H. Richardson , J. Hutchinson , C. A. Hunter , J. Phys. Chem. B 2010, 114, 11697–11702. 20735119 10.1021/jp102755h

[67] M. Evyapan , A. K. Hassan , A. D. F. Dunbar , Sens. Actuators B 2018, 254, 669–680.

[68] S. Kladsomboona , T. Kerdcharoen , Anal. Chim. Acta 2011, 757, 75–82. 10.1016/j.aca.2012.10.054 23206399

[69] J. Long , J. Xu , Y. Yang , J. Wen , C. Jia , Mater. Sci. Eng. C 2011, 25, 1271–1276.

[70a] D. Filippini , A. Alimelli , C. Di Natale , R. Paolesse , A. D′Amico , I. Lundström , Angew. Chem. Int. Ed. 2006, 45, 3800–3803; 10.1002/anie.200600050 16671131

[70b] P. Preechaburana , A. Suska , D. Filippini , Trends Biotechnol. 2014, 32, 351–355. 24702730 10.1016/j.tibtech.2014.03.007

[71] T. W. Bell , N. M. Hext , Chem. Soc. Rev. 2004, 33, 589–598. 15592624 10.1039/b207182g

[72] J. Prabphal , T. Vilaivan , T. Praneenararat , ChemistrySelect 2018, 3, 894–899.

[73] X. Jiang , F. Geng , Y. Wang , J. Liu , P. Qu , M. Xu , Biosens. Bioelectron. 2016, 81, 268–273. 26971272 10.1016/j.bios.2016.02.068

[74] A. Garcia-Sampedro , A. Tabero , I. Mahamed , P. Acedo , J. Porphyrins Phthalocyanines 2019, 23, 11–27.

[75] R. I. Dmitriev , D. B. Papkovsky , Cell. Mol. Life Sci. 2012, 69, 2025–2039. 22249195 10.1007/s00018-011-0914-0 PMC3371327

[76] A. L. Harris , Nat. Rev. Cancer 2002, 2, 38–47. 11902584 10.1038/nrc704

[77a] M. Garcia-Diaz , Y.-Y. Huang , M. R. Hamblin , Methods 2016, 109, 158–166; 27374076 10.1016/j.ymeth.2016.06.025 PMC5075498

[77b] M. O. Senge , M. W. Radomski , Photodiagn. Photodyn. Ther. 2013, 10, 1–16. 10.1016/j.pdpdt.2012.08.004 23465367

[78] Y. Yabuki , Y. Iwamoto , K. Tsukada , TrAC Trends Anal. Chem. 2010, 29, 319–338.

[79a] I. Dunphy , S. A. Vinogradov , D. F. Wilson , Anal. Biochem. 2002, 310, 191–198; 12423638 10.1016/s0003-2697(02)00384-6

[79b] R. P. Briñas , T. Troxler , R. M. Hochstrasser , S. A. Vinogradov , J. Am. Chem. Soc. 2005, 127, 11851–11862; 16104764 10.1021/ja052947c PMC2441878

[79c] O. S. Finikova , A. Y. Lebedev , A. Aprelev , T. Troxler , F. Gao , C. Garnacho , S. Muro , R. M. Hochstrasser , S. A. Vinogradov , ChemPhysChem 2008, 9, 1673–1679; 18663708 10.1002/cphc.200800296 PMC2645351

[79d] A. Y. Lebedev , A. V. Cheprakov , S. Sakadžić , D. A. Boas , D. F. Wilson , S. A. Vinogradov , ACS Appl. Mater. Interfaces 2009, 1, 1292–1304; 20072726 10.1021/am9001698 PMC2805241

[79e] J. A. Spencer , F. Ferraro , E. Roussakis , A. Klein , J. Wu , J. M. Runnels , W. Zaher , L. J. Mortensen , C. Alt , R. Turcotte , R. Yusuf , D. Côté , S. A. Vinogradov , D. T. Scadden , C. P. Lin , Nature 2014, 508, 269–273. 24590072 10.1038/nature13034 PMC3984353

[80] A. Fercher , G. V. Ponomarev , D. Yashunski , D. Papkovsky , Anal. Bioanal. Chem. 2010, 396, 1793–1803. 20063150 10.1007/s00216-009-3399-z

[81a] R. I. Dmitriev , A. V. Zhdanov , G. V. Ponomarev , D. V. Yashunski D B Papkovsky , Anal. Biochem. 2010, 398, 24–33; 19931212 10.1016/j.ab.2009.10.048

[81b] R. I. Dmitriev , H. M. Ropiak , D. V. Yashunsky , G. V. Ponomarev , A. V. Zhdanov , D. B. Papkovsky , FEBS J. 2010, 277, 4651–4661; 20883447 10.1111/j.1742-4658.2010.07872.x

[81c] R. I. Dmitriev , H. M. Ropiak , G. V. Ponomarev , D. V. Yashunsky , D. B. Papkovsky , Bioconjugate Chem. 2011, 22, 2507–2518. 10.1021/bc200324q 22035070

[82] R. I. Dmitriev , N. O′Donnell , D. B. Papkovsky , Bioconjugate Chem. 2016, 27, 439–445. 10.1021/acs.bioconjchem.5b00535 26704593

[83] K. Koren , R. I. Dmitriev , S. M. Borisov , D. B. Papkovsky , I. Klimant , ChemBioChem 2012, 13, 1184–1190. 22532338 10.1002/cbic.201200083 PMC3437475

[84] P. W. Zach , O. T. Hofmann , I. Klimant , S. M. Borisov , Anal. Chem. 2018, 90, 2741–2748. 29376644 10.1021/acs.analchem.7b04760

[85] B. Nacht , C. Larndorfer , S. Sax , S. M. Borisov , M. Hajnsek , F. Sinner , E. J. W. List-Kratochvil , I. Klimant , Biosens. Bioelectron. 2015, 64, 102–110. 25194803 10.1016/j.bios.2014.08.012

[86] N. P. Kamat , Z. Liao , L. E. Moses , J. Rawson , M. J. Therien , I. J. Dmochowski , D. A. Hammer , Proc. Natl. Acad. Sci. USA 2011, 108, 13984–13989. 21844376 10.1073/pnas.1102125108 PMC3161589

[87] Y. Zang , J. Lei , H. Ju , Biosens. Bioelectron. 2017, 96, 8–16. 28454070 10.1016/j.bios.2017.04.030

[88] W. Tu , Y. Dong , J. Lei , H. Ju , Anal. Chem. 2010, 82, 8711–8716. 20857916 10.1021/ac102070f

[89] W. Tu , J. Lei , P. Wang , H. Ju , Chem. Eur. J. 2011, 17, 9440–9447. 21678510 10.1002/chem.201100577

[90] G. Magna , Y. Sivalingam , E. Martinelli , G. Pomarico , F. Basoli , R. Paolesse , C. D. Natale , Anal. Chim. Acta 2014, 810, 86–93. 24439509 10.1016/j.aca.2013.12.008

[91a] T. P. Dalton , H. G. Shertzer , A. Puga , Annu. Rev. Pharmacol. Toxicol. 1999, 39, 67–101; 10331077 10.1146/annurev.pharmtox.39.1.67

[91b] P. Schröder , D. Daubner , H. Maier , J. Neustifter , R. Debus , Bioresour. Technol. 2008, 99, 7183–7191. 18313917 10.1016/j.biortech.2007.12.081

[92] X. Zhu , H. Li , D. Zhang , W. Chen , M. Fu , S. Nie , Y. Gao , Q. Liu , ACS Sustainable Chem. Eng. 2019, 7, 18105–18113.

[93] J. Shu , Z. Qiu , J. Zhuang , M. Xu , D. Tang , ACS Appl. Mater. Interfaces 2015, 7, 23812–23818. 26451956 10.1021/acsami.5b08742

[94] W.-W. Zhao , L. Zhang , J.-J. Xu , H.-Y. Chen , Chem. Commun. 2012, 48, 9456–9458. 10.1039/c2cc34543a 22820862

[95] P. Zhu , P. Wang , L. Kan , G. Sun , Y. Zhang , J. Yu , Biosens. Bioelectron. 2015, 68, 604–610. 25643601 10.1016/j.bios.2015.01.050

[96] X. Ma , J. Chen , Y. Wu , S. Devaramani , X. Hu , Q. Niu , C. Zhang , D. Shan , H. Wang , X. Lu , J. Electroanal. Chem. 2018, 820, 123–131.

[97] G.-Y. Zhang , Y.-H. Zhuang , D. Shan , G.-F. Su , S. Cosnier , X.-J. Zhang , Anal. Chem. 2016, 88, 11207–11212. 27750417 10.1021/acs.analchem.6b03484

[98] Q. Hong , L. Ge , W. Wang , X. Liu , F. Li , Biosens. Bioelectron. 2018, 121, 90–95. 30199713 10.1016/j.bios.2018.08.071

[99] E. Sitte, M. O. Senge, *Eur. J. Org. Chem* **2020**, 10.1002/ejoc.202000074. PMC7319466 32612451

[100] Axial ligand(s) not specified by the authors.

[101] J. Li , J. Lei , Q. Wang , P. Wang , H. Ju , Electrochim. Acta 2012, 83, 73–77.

[102] Y. Zang , J. Lei , P. Ling , H. Ju , Anal. Chem. 2015, 87, 5430–5436. 25902380 10.1021/acs.analchem.5b00888

[103] M. Li , X. Tian , W. Liang , R. Yuan , Y. Chai , Anal. Chem. 2018, 90, 14521–14526. 30444350 10.1021/acs.analchem.8b04370

[104a] J. Wang , Chem. Rev. 2008, 108, 814–825; 18154363 10.1021/cr068123a

[104b] M.-S. Steiner , A. Duerkop , O. S. Wolfbeis , Chem. Soc. Rev. 2011, 40, 4805–4839; 21674076 10.1039/c1cs15063d

[104c] A. L. Galant , R. C. Kaufman , J. D. Wilson , Food Chem. 2015, 188, 149–160; 26041177 10.1016/j.foodchem.2015.04.071

[104d] H.-C. Wang , A.-R. Lee , ScienceDirect 2015, 23, 191–200;

[104e] S. A. Zadi , J. H. Shin , Talanta 2016, 149, 30–42. 26717811 10.1016/j.talanta.2015.11.033

[105] H. Wei , E. Wang , Anal. Chem. 2008, 80, 2250–2254. 18290671 10.1021/ac702203f

[106] L. Gao , J. Zhuang , L. Nie , J. Zhang , Y. Zhang , N. Gu , T. Wang , J. Feng , D. Yang , S. Perrett , X. Yan , Nat. Nanotechnol. 2015, 3, 13254–13262. 10.1038/nnano.2007.260 18654371

[107] D. Feng , Z.-Y. Gu , J.-R. Li , H.-L. Jiang , Z. Wei , H.-C. Zhou , Angew. Chem. Int. Ed. 2012, 51, 10307–10310; 10.1002/anie.201204475 22907870

[108] Q. Liu , H. Li , Q. Zhao , R. Zhu , Y. Yang , Q. Jia , B. Bian , L. Zhuo , Mater. Sci. Eng. C 2014, 41, 142–151. 10.1016/j.msec.2014.04.038 24907747

[109] Q. Liu , Y. Yang , H. Li , R. Zhu , Q. Shao , S. Yang , J. Xu , Biosens. Bioelectron. 2015, 64, 147–153. 25212068 10.1016/j.bios.2014.08.062

[110a] Q. Liu , Y. Ding , Y. Yang , L. Zhang , L. Sun , P. Chen , C. Gao , Mater. Sci. Eng. C 2016, 59, 445–453; 10.1016/j.msec.2015.10.046 26652395

[110b] Q. Liu , Y. Yang , X. Lv , Y. Ding , Y. Zhang , J. Jing , C. Xu , Sens. Actuators B 2017, 240, 726–734.

[111] Q. Liu , R. Zhu , H. Du , H. Li , Y. Yang , Q. Jia , B. Bian , Mater. Sci. Eng. C 2014, 43, 321–329. 10.1016/j.msec.2014.07.032 25175220

[112] Q. Liu , L. Zhang , H. Li , Q. Jia , Y. Jiang , Y. Yang , R. Zhu , Mater. Sci. Eng. C 2015, 55, 193–200. 10.1016/j.msec.2015.05.028 26117755

[113] Q. Liu , Q. Jia , R. Zhu , Q. Shao , D. Wang , P. Cui , J. Ge , Mater. Sci. Eng. C 2014, 42, 177–184. 10.1016/j.msec.2014.05.019 25063108

[114] Q. Liu , P. Chen , Z. Xu , M. Chen , Y. Ding , K. Yue , J. Xu , Sens. Actuators B 2017, 251, 339–348.

[115] B. Bing , Q. Liu , S. Yu , New J. Chem. 2018, 42, 18189–18200.

[116] Z. Xu , X. Lyu , B. Yang , W. Cao , R. Li , X. Zhang , X. Zhang , G. Fan , X. Kong , Q. Liu , Colloids Surf. A 2019, 569, 28–34.

[117] Y. Yang , F. Tan , X. Xie , X. Yang , Z. Zhou , K. Deng , H. Huang , Anal. Sci. 2019, 35, 691–699. 30853695 10.2116/analsci.19P004

[118a] M. Aghayan , A. Mahmoudi , K. Nazari , S. Dehghanpour , S. Sohrabi , M. R. Sazegar , N. Mohammadian-Tabrizi , J. Porous Mater. 2019, 26, 1507–1521;

[118b] M. Aghayan , A. Mahmoudi , S. Sohrabi , S. Dehghanpour , K. Nazari , N. Mohammadian-Tabrizi , Anal. Methods 2019, 11, 3175–3187.

[119] T. Li , P. Hu , J. Li , P. Huang , W. Tong , C. Gao , Colloids Surf. A 2019, 577, 456–463.

[120a] T. Muller , S. Bräse , RSC Adv. 2014, 4, 6886–6907;

[120b] Z. Hassan , Y. Matt , S. Begum , M. Tsotsalas , S. Bräse , Adv. Funct. Mater. 2020, 1907625.

[121] C. Cui , Q. Wang , Q. Liu , X. Deng , T. Liu , D. Li , X. Zhang , Sens. Actuators B 2018, 277, 86–94.

[122] J. Wang , X. Yang , T. Wei , J. Bao , Q. Zhu , Z. Dai , ACS Appl. Bio Mater. 2018, 1, 382–388. 10.1021/acsabm.8b00104 35016394

[123] T. Liu , J. Tian , L. Cui , Q. Liu , L. Wu , X. Zhang , Colloid. Surf. B 2019, 178, 137–145. 10.1016/j.colsurfb.2019.03.008 30852265

[124] X. Fan , R. Tian , T. Wang , S. Liu , L. Wang , J. Xu , J. Liu , M. Ma , Z. Wu , Nanoscale 2018, 10, 22155–22160. 30474099 10.1039/c8nr07288d

[125] X. Wang , B. Xu , Z. Liu , J. Mater. Sci. 2018, 53, 8786–8794.

[126] S. Bhunia , N. Dey , A. Pradhan , S. Bhattacharya , Chem. Commun. 2018, 54, 7495–7498. 10.1039/c8cc02865f 29922790

[127] G. Pandey , R. Chaudhari , B. Joshi , S. Choudhary , J. Kaur , A. Joshi , Sci. Rep. 2019, 9, 5029. 30903010 10.1038/s41598-019-41326-7 PMC6430792

[128a] L. R. Bornhoeft , A. Biswas , M. J. McShane , Biosensors 2017, 7, 8; 10.3390/bios7010008 PMC5371781 28117762

[128b] B. Khurana , P. Gierlich , A. Meindl , L. C. Gomes-da-Silva , M. O. Senge , Photochem. Photobiol. Sci. 2019, 18, 2613–2656. 31460568 10.1039/c9pp00221a

[129] B. J. van Enter , E. von Hauff , Chem. Commun. 2018, 54, 5032–5045. 10.1039/c8cc01678j 29687110

[130] Y. Zhang , Y. Guo , Y. Xianyu , W. Chen , Y. Zhao , X. Jiang , Adv. Mater. 2013, 25, 3802–3819. 23740753 10.1002/adma.201301334

[131a] L. M. Moreira , A. L. Poli , J. P. Lyon , F. Aimbire , J. C. Toledo Jr. , A. J. C. -Filho , H. Imasato , J. Porphyrins Phthalocyanines 2010, 14, 199-218;

[131b] E. H. S. Sousa , L. G. D. F. Lopes , G. Gonzalez , M.-A. Gilles-Gonzalez , J. Inorg. Biochem. 2017, 167, 12–20. 27893989 10.1016/j.jinorgbio.2016.11.022

[132a] E. Schneider , D. S. Clark , Biosens. Bioelectron. 2013, 39, 1–13; 22809523 10.1016/j.bios.2012.05.043

[132b] A. Yarman , U. Wollenberger , F. W. Scheller , Electrochim. Acta 2013, 110, 63–72.

[133a] M. B. Winter , E. J. McLaurin , S. Y. Reece , C. Olea , D. G. Nocera , M. A. Marletta , J. Am. Chem. Soc. 2010, 132, 5582–5583; 20373741 10.1021/ja101527r PMC2859244

[133b] M. H. Vos , L. Bouzhir-Sima , J.-C. Lambry , H. Luo , J. J. Eaton-Rye , A. Ioanoviciu , P. R. Ortiz de Montellano , U. Liebl , Biochemistry 2012, 51, 159–166; 22142262 10.1021/bi201467c PMC3254832

[133c] T. Itoh , S. -i Matsuura , T. T. Chuong , O. Tanaike , S. Hamakawa , T. Shimizu , Anal. Sci. 2019, 35, 329–335. 30449836 10.2116/analsci.18P449

[134] Z. Dai , E. M. Boon , J. Am. Chem. Soc. 2010, 132, 11496–11503. 20684546 10.1021/ja101674z

[135a] S. Muralidharan , E. M. Boon , J. Am. Chem. Soc. 2012, 134, 2044–2046; 22257139 10.1021/ja211576b

[135b] Y. Li , Q. Liu , X. Liang , Q. Xiao , Y. Fang , Y. Wu , Sens. Actuators B 2016, 230, 405–410;

[135c] B. Gong X Liang , Y. Li , Q. Xiao , P. Yang , Y. Wu , Appl. Biochem. Biotechnol. 2018, 184, 102–112. 28624997 10.1007/s12010-017-2535-z

[136] P. A. Sigala , K. Morante , K. Tsumoto , J. M. M. Caaveiro , D. E. Goldberg , Biochemistry 2016, 55, 4836–4849. 27490825 10.1021/acs.biochem.6b00562 PMC5007156

[137] Y. Pan , Y. Yang , Y. Pang , Y. Shi , Y. Long , H. Zheng , Talanta 2018, 185, 433–438. 29759224 10.1016/j.talanta.2018.04.005

[138] M.-L. Hong , L.-J. Li , H.-X. Han , X. Chu , Anal. Sci. 2011, 30, 811–815. 10.2116/analsci.30.811 25109643

[139] W. R. Edwards , A. J. Williams , J. L. Morris , A. J. Baldwin , R. K. Allemann , D. D. Jones , Biochemistry 2010, 49, 6541–6549. 20602528 10.1021/bi100793y

[140] C. L. Nobles , J. R. Clark , S. I. Green , A. W. Maresso , J. Microbiol. Methods 2015, 118, 7–17. 26253803 10.1016/j.mimet.2015.07.011 PMC5837058

[141] J. E. Hyeon , D. W. Jeong , Y. J. Ko , S. W. Kim , C. Park , S. O. Han , Biosens. Bioelectron. 2018, 114, 1–9. 29775852 10.1016/j.bios.2018.05.007

[142] M. Lian , S. Zhang , J. Chen , X. Liu , X. Chen , W. Yang , ACS Appl. Bio Mater. 2019, 2, 2185–2191. 10.1021/acsabm.9b00160 35030657

[143] V. Villari , P. Mineo , E. Scamporrino , N. Micali , Chem. Phys. 2012, 402, 118–123.

[144] K. Konopińska , M. Pietrzak , E. Malinowska , Microchem. J. 2014, 115, 1–5.

[145] K. Konopińska , M. Pietrzak , E. Malinowska , Anal. Biochem. 2015, 470, 41–47. 25447459 10.1016/j.ab.2014.09.024

[146] Y. D. Ivanov , T. O. Pleshakova , N. V. Krohin , A. L. Kaysheva , S. A. Usanov , A. I. Archakov , Biosens. Bioelectron. 2013, 84, 71–77. 10.1016/j.bios.2012.12.039 23357004

[147] X. Chen , Y.-W. Wu , Org. Biomol. Chem. 2016, 14, 5417–5439. 26940577 10.1039/c6ob00126b

[148] L. Yang , J. Han , W. Liu , J. Li , L. Jiang , Anal. Chem. 2015, 87, 5270–5277. 25921798 10.1021/acs.analchem.5b00376

[149] B. J. Johnson , C. R. Taitt , A. Gleaves , S. H. North , A. P. Malanoski , I. A. Leska , E. Archibong , S. M. Monk , Sens. Biosens. Res. 2016, 8, 1–7.

[150] M. D. Frank-Kamenetskii , Phys. Rep. 1997, 288, 13–60.

[151a] R. F. Pasternack , E. J. Gibbs , J. J. Villafranca , Biochemistry 1983, 22, 5409–5417; 6652071 10.1021/bi00292a024

[151b] R. J. Fiel , J. Biomol. Struct. Dyn. 1989, 6, 1259–1274; 2684219 10.1080/07391102.1989.10506549

[151c] L. A. Lipscomb , F. X. Zhou , S. R. Presnell , R. J. Woo , M. E. Peek , R. R. Plaskon , L. D. Williams , Biochemistry 1996, 35, 2818–2823. 8608116 10.1021/bi952443z

[152] E. Vaishnavi , R. Renganathan , Analyst 2014, 139, 225–234. 24187682 10.1039/c3an01871g

[153] F. Lin , Y. Zhou , Q. Li , X. Zhou , Y. Shao , B. Habermeyer , H. Wang , X. Shi , Z. Xu , Anal. Chem. 2017, 89, 9299–9306. 28738682 10.1021/acs.analchem.7b02077

[154a] M. Balaz , M. De Napoli , A. E. Holmes , A. Mammana , K. Nakanishi , N. Berova , R. Purrello , Angew. Chem. Int. Ed. 2005, 44, 4006–4009; 10.1002/anie.200501149 15915533

[154b] A. D'Urso , A. Mammana , M. Balaz , A. E. Holmes , N. Berova , R. Lauceri , R. Purrello , J. Am. Chem. Soc. 2009, 131, 2046–2047. 19159291 10.1021/ja808099u

[155] A. D′Urso , A. E. Holmes , N. Berova , M. Balaz , R. Purrello , Chem. Asian J. 2011, 6, 3104–3109. 21882349 10.1002/asia.201100161

[156] J. K. Choi , G. Sargsyan , M. S. -Hussain , A. E. Holmes , M. Balaz , J. Phys. Chem. B 2011, 115, 10182–10188. 21774503 10.1021/jp2047213 PMC3177531

[157] J. Xu , J. Wu , C. Zong , H. Ju , F. Yan , Anal. Chem. 2013, 85, 3374–3379. 23427829 10.1021/ac4000688

[158] N. Xu , J. Lei , Q. Wang , Q. Yang , H. Ju , Talanta 2016, 150, 661–665. 26838456 10.1016/j.talanta.2016.01.005

[159] D. Sen , L. C. H. Poon , Crit. Rev. Biochem. Mol. Biol. 2011, 46, 478–492. 21958168 10.3109/10409238.2011.618220

[160] C. E. Pearson , R. R. Sinden , Curr. Opin. Struct. Biol. 1998, 8, 321–330. 9666328 10.1016/s0959-440x(98)80065-1

[161a] D.-L. Ma , D. S.-H. Chan , H. Yang , H.-Z. He , C.-H. Leung , Curr. Pharm. Des. 2012, 18, 2058–2075; 22376110 10.2174/138161212799958314

[161b] O. Doluca , J. M. Whiters , V. V. Filichev , Chem. Rev. 2013, 113, 3044–3083. 23391174 10.1021/cr300225q

[162] B. Ruttkay-Nedecky , J. Kudr , L. Nejdl , D. Maskova , R. Kizek , V. Adam , Molecules 2013, 18, 14760–14779. 24288003 10.3390/molecules181214760 PMC6270327

[163] L.-M. Zhang , Y.-X. Cui , L.-N. Zhu , J.-Q. Chu , D.-M. Kong , Nucleic Acids Res. 2019, 47, 2727–2738. 30715502 10.1093/nar/gkz064 PMC6451126

[164] Y.-F. Huo , L.-N. Zhu , K.-K. Liu , L.-N. Zhang , R. Zhang , D.-M. Kong , Inorg. Chem. 2017, 56, 6330–6342. 28474900 10.1021/acs.inorgchem.7b00426

[165] L.-N. Zhang , R. Zhang , Y.-X. Cui , K.-K. Liu , D.-M. Kong , X.-Z. Li , L.-N. Zhu , Dyes Pigm. 2017, 145, 404–417.

[166] Y.-F. Huo , L.-N. Zhu , X.-Y. Li , G.-M. Han , D.-M. Kong , Sens. Actuators B 2016, 237, 179–189.

[167] R. Zhang , M. Cheng , L.-M. Zhang , L.-N. Zhu , D.-M. Kong , ACS Appl. Mater. Interfaces 2018, 10, 13350–13360. 29619818 10.1021/acsami.8b01901

[168a] I. Willner , B. Shlyahovsky , M. Zayats , B. Willner , Chem. Soc. Rev. 2008, 37, 1153–1165; 18497928 10.1039/b718428j

[168b] H. Yaku , T. Murashima , D. Miyoshi , N. Sugimoto , Molecules 2012, 17, 10586–10613; 22951397 10.3390/molecules170910586 PMC6268517

[168c] J. L. Neo , K. Kamaladasan , M. Uttamchandani , Curr. Pharm. 2012, 18, 2048–2057; 10.2174/138161212799958341 22380516

[168d] Y. Fu , X. Wang , J. Zhang , W. Li , Curr. Opin. Biotechnol. 2014, 28, 33–38; 24832072 10.1016/j.copbio.2013.10.014

[168e] H. Funabashi , Electrochemistry 2016, 84, 290–295;

[168f] A.-M. Chiorcea-Paquim , A. M. Oliveira-Brett , Chemosensors 2016, 4, 13–33;

[168g] X. Cui , R. Li , X. Liu , J. Wang , X. Leng , X. Song , Q. Pei , Y. Wang , S. Liu , J. Huang , Anal. Chim. Acta 2018, 997, 1–8; 29149989 10.1016/j.aca.2017.10.009

[168h] M. Mahdiannasser , Z. Karami , Biosens. Bioelectron. 2018, 107, 123–144; 29455023 10.1016/j.bios.2018.02.020

[168i] J. H. Yum , S. Park , H. Sugiyama , Org. Biomol. Chem. 2019, 17, 9547–9561; 31670331 10.1039/c9ob01876j

[168j] Z.-L. Liu , C.-A. Tao , J.-F. Wang , Chin. J. Anal. Chem. 2020, 48, 153–163.

[169] L. Zhang , J. Zhu , J. Ai , Z. Zhou , X. Jia , E. Wang , Biosens. Bioelectron. 2013, 39, 268–273. 22921949 10.1016/j.bios.2012.07.058

[170] Q. Zhu , L. Liu , Y. Xing , X. Zhou , J. Hazard. Mater. 2018, 355, 50–55. 29772375 10.1016/j.jhazmat.2018.04.082

[171] X. Zhang , B. Ding , H. Wu , J. Wang , H. Yang , Anal. Sci. 2017, 33, 165–169. 28190835 10.2116/analsci.33.165

[172] Q. Chen , Q. Guo , Y. Chen , J. Pang , F. Fu , L. Guo , Talanta 2015, 138, 15–19. 25863365 10.1016/j.talanta.2015.02.002

[173] J. Sun , W. Jiang , J. Zhu , W. Li , L. Wang , Biosens. Bioelectron. 2015, 70, 15–20. 25775969 10.1016/j.bios.2015.03.014

[174] J.-L. He , Y. Zhang , C. Yang , S.-Y. Huang , L. Wu , T.-T. Mei , J. Wang , Z. Cao , Anal. Methods 2019, 11, 2204–2210.

[175] S. M. Taghdisi , N. M. Danesh , M. Ramezani , K. Abnous , Food Chem. 2018, 268, 342–346. 30064767 10.1016/j.foodchem.2018.06.101

[176] Y. Li , S. Liu , Z. Zhao , Y. Zheng , Z. Wang , Talanta 2017, 164, 196–200. 28107917 10.1016/j.talanta.2016.11.047

[177a] T. Li , E. Wang , S. Dong , Anal. Chem. 2010, 82, 7576–7580; 20726508 10.1021/ac1019446

[177b] J. Ren , H. Qin , J. Wang , N. W. Luedtke , E. Wang , J. Wang , Anal. Bioanal. Chem. 2011, 399, 2763–2770; 21267713 10.1007/s00216-011-4669-0

[177c] Q. Yue , T. Shen , C. Wang , L. Wang , H. Lia , S. Xu , H. Wang , J. Liu , Biosens. Bioelectron. 2013, 40, 75–81. 22794935 10.1016/j.bios.2012.06.026

[178] R. Cheng , M. Tao , Z. Shi , X. Zhang , Y. Jin , B. Li , Biosens. Bioelectron. 2015, 73, 138–145. 26057733 10.1016/j.bios.2015.05.059

